# Epigallocatechin activates haem oxygenase-1 expression via protein kinase Cδ and Nrf2

**DOI:** 10.1016/j.bbrc.2008.06.068

**Published:** 2008-09-05

**Authors:** Richard M. Ogborne, Stuart A. Rushworth, Maria A. O’Connell

**Affiliations:** a MRC Human Nutrition Research, Fulbourn Road, Cambridge CB1 9NL, UK; b School of Chemical Sciences and Pharmacy, University of East Anglia, Norwich NR4 7TJ, UK

**Keywords:** Epigallocatechin, Haem oxygenase-1, Monocytic cells, Nrf2, Protein kinase C, Green tea polyphenols

## Abstract

The Nrf2/anti-oxidant response element (ARE) pathway plays an important role in regulating cellular anti-oxidants, including haem oxygenase-1 (HO-1). Various kinases have been implicated in the pathways leading to Nrf2 activation. Here, we investigated the effect of epigallocatechin (EGC) on ARE-mediated gene expression in human monocytic cells. EGC time and dose dependently increased HO-1 mRNA and protein expression but had minimal effect on expression of other ARE-regulated genes, including NAD(P)H:quinone oxidoreductase 1, glutathione cysteine ligase and ferritin. siRNA knock down of Nrf2 significantly inhibited EGC-induced HO-1 expression. Furthermore, inhibition of PKC by Ro-31-8220 dose dependently decreased EGC-induced HO-1 mRNA expression, whereas MAP kinase and phosphatidylinositol-3-kinase pathway inhibitors had no significant effect. EGC stimulated phosphorylation of PKCαβ and δ in THP-1 cells. PKCδ inhibition significantly decreased EGC-induced HO-1 mRNA expression, whereas PKCα- and β-specific inhibitors had no significant effect. These results demonstrate for the first time that EGC-induced HO-1 expression occurs via PKCδ and Nrf2.

Catechins, a family of plant polyphenols, exert anti-oxidant, anti-inflammatory and anti-proliferative effects in vitro and in vivo [1,2]. This may be in part due to their activation of Phase II enzymes and cellular anti-oxidants, including NAD(P)H:quinone oxidoreductase 1 (NQO1), catalase and enzymes involved in glutathione synthesis [3–5]. These and other cytoprotective molecules, including haem oxygenase-1 (HO-1, the rate-limiting enzyme in haem catabolism) and the iron-binding protein ferritin, contain anti-oxidant response elements (ARE) in the regulatory regions of their genes. The transcription factor NF-E2-related factor 2 (Nrf2) binds to this regulatory element and plays a key role in ARE-driven gene transcription [6]. Nrf2 knockout mice are deficient in cellular anti-oxidants and are susceptible to inflammation [7,8], demonstrating the importance of this pathway in cellular defence. Several kinase pathways have been implicated in activation of Nrf2 including the ERK and p38 MAP kinase pathways, phosphatidyl inositol 3 kinase and protein kinase C (PKC) [9–14].

Green tea is a rich source of catechins, comprising over 30% of its dry weight [15]. Green tea extract activates ARE-mediated reporter activity [16]. EGCG is the most abundant catechin in green tea and studies to date have focused on this compound. EGCG stimulates expression of many Nrf2-dependent genes in liver and intestine in mice and activates HO-1 expression in B lymphoblasts, epithelial and endothelial cells [13,14,17,18]. Epigallocatechin (EGC) is also a major catechin in green tea [19]. Although EGCG and EGC are structurally similar except for the addition of a 3-gallate group in EGCG, their biological effects differ extensively [3,20–26]. EGC is also more bioavailable than EGCG and other catechins and oral administration of EGC, but not EGCG, results in a significant increase in plasma anti-oxidant activity [27].

Monocytes play an essential role in the host response to oxidative stress and inflammation. In the present study, the effects of EGC on ARE-mediated gene expression were examined in THP-1 cells, a human monocytic cell line that we have previously used to examine ARE-mediated gene expression [9–11]. In addition, the role of Nrf2 and the various potential kinase pathways leading to Nrf2 activation were investigated.

## Materials and methods

*Materials.* LY333531 was purchased from Alexis Biotechnologies (Nottingham, UK). SB203580, Ro-31-8220, rottlerin, LY294002, Go6976 and PD98059 were obtained from Calbiochem (Nottingham, UK). (−)Epigallocatechin and all other chemicals were purchased from Sigma (Poole, UK).

*Cell culture.* THP-1, a human monocytic leukaemia cell line [28], was purchased from ECACC (Porton Down, UK) and cultured in RPMI 1640 medium supplemented with 10% foetal calf serum, 2 mM l-glutamine (Biowhittaker, Wokingham, UK) and 2-mercaptoethanol. Cells were maintained in a humidified atmosphere at 37 °C and 5% CO_2_.

*Western immunoblotting.* Cells (1 × 10^6^) were unstimulated or stimulated with EGC and whole cell lysates prepared, proteins separated and immunoblotting carried out as previously described [11]. Antibodies were purchased from the following: mouse anti-human HO-1 antibody (Stressgen Biotechnologies Corporation, Victoria, Canada); rabbit anti-human phosphorylated PKCαβ and PKCδ antibodies (Cell Signalling Technology, Beverley, USA); mouse anti-human endogenous PKCδ antibody (BD Biosciences, CA, USA); goat anti-mouse and goat anti-rabbit secondary antibodies (Santa Cruz Biotechnology, Santa Cruz, USA); mouse anti-human β-actin antibody (Sigma).

*Real-time PCR.* Cells (5 × 10^5^) were unstimulated or stimulated with EGC for various times at 37 °C. In some experiments, cells were pre-treated with kinase inhibitors for 30 min prior to EGC stimulation. RNA extraction, reverse transcription, and real-time PCR were carried out as previously described [10]. Relative quantitative mRNA expression of HO-1, NQO1, GCLM or ferritin was normalized to 18s ribosomal unit mRNA expression.

*Nrf2 siRNA transfection.* Nrf2 siRNA sense sequences 5′-GAGUAUGAGCUGGAAAAACtt-3′ (siNrf2 A) [29], 5′-CCUUAUAUCUCGAAGUUUUtt-3′ (siNrf2 B), their complementary antisense sequences and negative controls were obtained from Ambion as purified annealed duplexes. THP-1 cells (5 × 10^4^/well) were transfected in serum-free media with control siRNA or Nrf2-targeted siRNA (30 nM final concentration), using Oligofectamine transfection reagent according to the manufacturer’s instructions (Invitrogen). Transfected cells were incubated for 48 h, with addition of 10% FCS at 4 h. Following this, cells were stimulated with EGC for 4 h before RNA extraction and real-time PCR analysis.

*PKCδ antisense oligodeoxyribonucleotide (ODN) transfection.* THP-1 cells (5 × 10^4^) were transfected in serum-free media with sense or antisense ODN to PKCδ, using Oligofectamine transfection reagent (Invitrogen), as previously described [10]. Following transfection, cells were unstimulated or stimulated with EGC for 4 h, total RNA extracted and real-time PCR performed.

*Statistical analyses.* Where indicated, statistical analyses were performed using paired Student’s *t* test. Results are means ± SD of three independent experiments. Results with *p* < 0.05 were considered statistically significant.

## Results

### EGC increases HO-1 expression in THP-1 cells

**Fig. 1:**
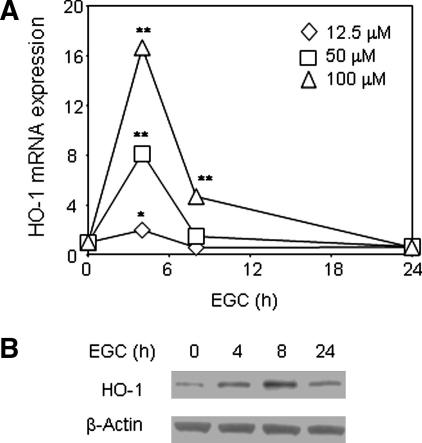
EGC increases HO-1 expression. (A) THP-1 cells were treated with 0–100 μM EGC for 4, 8 or 24 h. RNA was extracted, reverse transcribed and real-time PCR performed for HO-1 mRNA expression. Mean ± SD, three independent experiments. Student’s two-tailed *t* test, ^∗^*p* < 0.05, ^∗∗^*p* < 0.01. (B) THP-1 cells were treated with 100 μM EGC for indicated times, whole cell extracts prepared and Western blotting performed. Representative of three independent experiments.

EGC increased HO-1 mRNA expression in THP-1 cells, peaking at 4 h (*p* < 0.01), remaining elevated at 8 h (*p* < 0.01) and returning to baseline by 24 h (Fig. 1A). This correlated with an elevation in HO-1 protein expression by 4 h, which further increased by 8 h and decreased, but was still evident, at 24 h (Fig. 1B). EGC also time and dose dependently increased GCLM mRNA expression in THP-1 cells with maximal induction at 4 h. However, the induction was much weaker than that seen with HO-1 (GCLM at 4 h, 50 μM EGC, 2.5 ± 0.5, *p* < 0.01; 100 μM EGC, 3.0 ± 1.30, *p* < 0.01, mean fold increase above control ± SD, *n* = 7). EGC (12.5–100 μM) had no significant effect on either NQO1 or ferritin gene expression up to 24 h (data not shown). To ensure that EGC-induced HO-1 expression was not the result of toxicity, THP-1 cells pre-incubated with EGC for 24 h were analysed by MTT assay. At concentrations up to 125 μM THP-1 cells remained 96% (±2.3) viable compared to control cells, suggesting that the EGC concentrations used in this study did not exert cytotoxic effects.

### Role of Nrf2 in EGC-induced HO-1 expression

**Fig. 2:**
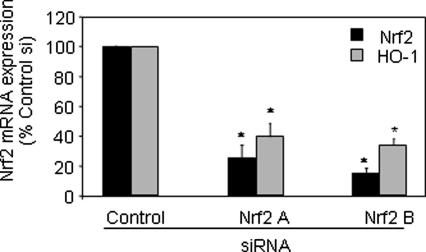
Nrf2 regulates EGC-induced HO-1 mRNA expression. THP-1 cells were transfected with control or Nrf2-targeted siRNA in serum-free media for 4 h followed by addition of 10% FCS for 44 h. Following this, cells were stimulated with EGC for 4 h before RNA extraction, reverse transcription and real-time PCR analysis for Nrf2 or HO-1 mRNA expression. Mean ± SD three independent experiments. Student’s two-tailed *t* test, ^∗^*p* < 0.05.

Nrf2 plays a key role in HO-1 regulation. The role of Nrf2 in EGC-induced HO-1 expression was confirmed by use of siRNA. THP-1 cells were transiently transfected with control or two different Nrf2 siRNAs and Nrf2 mRNA expression measured by real-time PCR. Fig. 2A demonstrates that both Nrf2 siRNAs significantly inhibited Nrf2 mRNA expression to a similar extent when compared with the negative control (black bars, *p* = 0.004, *p* = 0.0002, respectively). These siRNAs also inhibited Nrf2 protein expression (data not shown). In addition, they significantly inhibited EGC-induced HO-1 mRNA expression (grey bars, A, 60.7 ± 8.9, *p* = 0.01; B, 66.1 ± 2.6, *p* = 0.0007; mean ± SD% inhibition, *n* = 3), confirming that Nrf2 plays a key role in this pathway in THP-1 cells.

### Investigation of kinase pathways regulating EGC-induced HO-1 expression

**Fig. 3:**
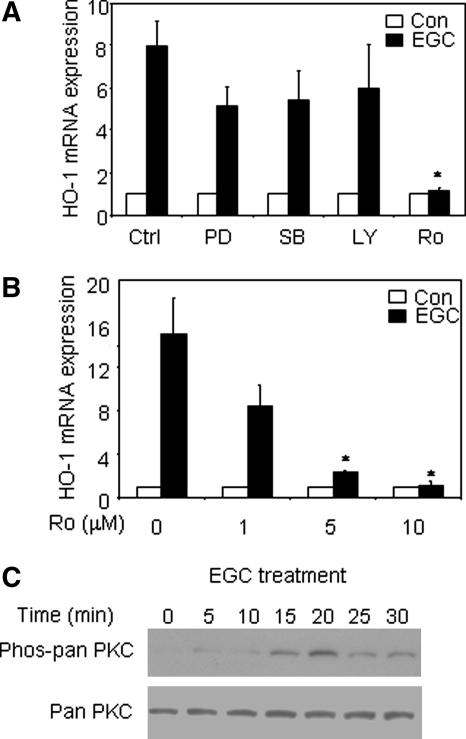
EGC-induced HO-1 mRNA expression is PKC-dependent. THP-1 cells were pre-treated with (A) 25 μM LY294002 (LY), 25 μM PD98059 (PD), 10 μM SB203580 (SB) or 5 μM Ro-31-8220 (Ro) or (B) varying concentrations of Ro-31-8220 for 30 min prior to stimulation with 100 μM EGC for 4 h. Total RNA was extracted, reverse transcribed and HO-1 mRNA expression detected by real-time PCR. Mean ± SD three independent experiments. Student’s two-tailed *t* test, ^∗^*p* < 0.05. (C) THP-1 cells were treated with 100 μM EGC for indicated times, whole cell extracts prepared and Western blotting performed. Representative of three independent experiments.

THP-1 cells were pre-treated with LY294002 (a PI3K inhibitor), SB203580 (a p38 MAPK inhibitor), PD98059 (an ERK MAPK pathway inhibitor) or Ro-31-8220 (a pan-PKC inhibitor) prior to stimulation with EGC. LY294002, SB203580 and PD98059 had no significant effect on EGC-induced HO-1 mRNA expression. However, pre-treatment with Ro-31-8220 abolished EGC-induced HO-1 mRNA expression (*p* < 0.05) (Fig. 3A), suggesting that this response is regulated by PKC. The effect of Ro-31-8220 upon EGC-induced HO-1 mRNA expression was concentration-dependent, confirming the specificity of this effect (Fig. 3B). Ro-31-8220 (5 μM) also completely abolished EGC-induced GCLM mRNA expression in these cells (data not shown). Furthermore, EGC induced phosphorylation of PKC within 5 min, peaked at 20 min (Fig. 3C) and remained phosphorylated at 4 and 8 h (data not shown). These results suggest that PKC plays a key role in EGC-induced HO-1 (and GCLM) expression in THP-1 cells.

### EGC-induced HO-1 expression is regulated by PKCδ

**Fig. 4:**
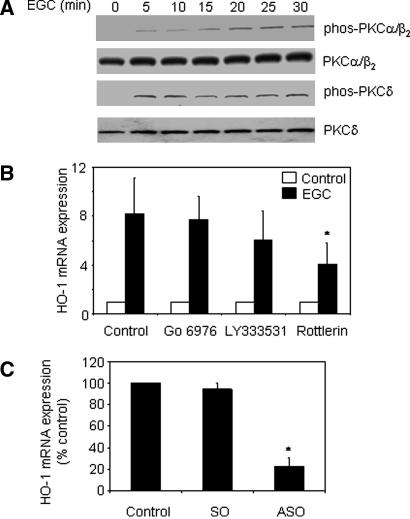
EGC-induced HO-1 expression is PKCδ-dependent. (A) THP-1 cells were treated with 100 μM EGC for indicated times, whole cell extracts prepared and Western blotting performed. Representative of three independent experiments. (B) Cells were pre-treated with 10 μM Go6976, 0.02 μM LY333531 or 15 μM Rottlerin for 30 min prior to stimulation with 100 μM EGC for 4 h. Total RNA was extracted, reverse transcribed and HO-1 mRNA expression detected by real-time PCR. Mean ± SD three independent experiments. Student’s two-tailed *t* test, ^∗^*p* < 0.05. (C) THP-1 cells were transfected with sense (SO) or antisense (ASO) ODN to PKCδ in serum-free media for 4 h followed by addition of 10% FCS for 44 h. Following transfection, cells were unstimulated or stimulated with indicated concentrations of EGC for 4 h, total RNA extracted, reverse transcribed and HO-1 mRNA expression detected by real-time PCR. Mean ± SD three independent experiments. Student’s two-tailed *t* test, ^∗^*p* < 0.05.

The PKC family of proteins comprises at least 10 serine/threonine kinases [30]. We have shown that LPS and curcumin-induced HO-1 expression in THP-1 cells are mediated via a classical PKC isoform and PKCδ, respectively [10,11]. The potential role of these PKC isoforms in regulating EGC-induced HO-1 expression in THP-1 cells was examined. EGC induced phosphorylation of classical PKCs(αβ) and PKCδ by 5 min, which was sustained at 30 min (Fig. 4A). This was not the result of a change in expression of unphosphorylated PKC isoforms. Cells were also pre-treated with LY333531 (a PKC β_1_/β_2_ inhibitor), Go6976 (a PKCα/β_1_ inhibitor) or rottlerin (which inhibits PKCδ and θ at the concentrations used) prior to stimulation with EGC and real-time PCR performed. LY333531 and Go6976 exerted no significant effect on EGC-induced HO-1 mRNA expression, suggesting that classical PKC isoforms are not important for this pathway. However, 15 μM rottlerin significantly inhibited EGC-induced HO-1 mRNA expression (*p* < 0.05) (Fig. 4B), suggesting that, similarly to curcumin, PKCδ may be important in HO-1 induction by EGC. Rottlerin, but not Go6976 or LY333531, also inhibited EGC-induced GCLM mRNA expression in THP-1 cells by 61% (data not shown). As rottlerin may also inhibit PKCθ and other kinase pathways, PKCδ antisense ODN were employed to confirm specificity. PKCδ antisense ODN inhibited PKCδ protein expression in THP-1 cells [10]. Transfection of PKCδ sense ODN did not affect EGC-induced HO-1 mRNA expression (Fig. 4C). However, transfection of antisense ODN prior to EGC stimulation resulted in a 78% reduction (*p* < 0.005) in EGC-induced HO-1 mRNA expression, confirming a role for PKCδ in the regulation of EGC-induced HO-1 expression.

## Discussion

Green tea extract activates ARE-dependent gene expression and studies to date have focused on EGCG. In the present study, EGC, a more bioavailable catechin found in green tea, induced HO-1 expression and GCLM expression in human monocytic THP-1 cells. Furthermore, EGC-induced HO-1 expression was regulated by Nrf2 and PKCδ.

We have previously reported the activation of Nrf2/ARE-mediated gene expression by dietary anti-oxidants in THP-1 cells. Alpha lipoic acid activates Nrf2-mediated HO-1 expression [9] and curcumin activates expression of Nrf2-regulated HO-1, NQO1, glutathione cysteine ligase and ferritin [10]. However, other dietary anti-oxidants including ascorbic acid, alpha tocopherol, gamma tocopherol and resveratrol do not activate these genes in THP-1 cells (unpublished). In the present study, EGC activated HO-1 and GCLM, but not NQO1 or ferritin expression in THP-1 cells. EGCG minimally increases HO-1 mRNA expression in THP-1 cells (unpublished). EGCG activates HO-1 mRNA and protein expression in epithelial and endothelial cells at similar concentrations used in our study [14,18]. However, in contrast to our results, 100 μM EGC had no effect on HO-1 protein expression in endothelial cells, which may be due to differences in cell type. Nrf2 regulates EGCG-induced HO-1 in B lymphoblasts and epithelial cells [13,18]. Here, EGC also activated Nrf2 in THP-1 cells, and Nrf2 silencing significantly suppressed EGC-induced HO-1 expression, suggesting a key role for this transcription factor in this pathway. However, EGC did not activate ARE-driven reporter activity in HepG2 cells transfected with an ARE reporter [3].

Small differences in catechin structure result in wide-ranging biological effects. The presence of a 3-gallate group in EGCG and epicatechin gallate (ECG) results in pharmacokinetic differences. EGC is more bioavailable than either EGCG or ECG and oral administration of EGC results in a higher plasma anti-oxidant activity than that seen with gallated catechins [27], suggesting that it may be more important in vivo. In addition, cellular effects differ between gallated and non-gallated catechins. EGCG and ECG, but not EGC, bind to oestrogen receptors in MCF7 cells [21]. However, in the same study, EGCG, but not EGC or the gallated ECG, activated oestrogen receptor-mediated gene expression. Catechins also exhibit varying cytotoxic effects in different cells [3,22]. In addition, EGCG, but not EGC, (i) binds to the metastasis-associated laminin receptor in tumour cells, (ii) disrupts liposome membrane structure, (iii) inhibits CYP450 isoforms, (iv) inhibits proteasome activity and (v) suppresses Type I collagen and matrix metalloproteinase 1 production [20,23–26].

Several kinase pathways may regulate EGC-induced HO-1 and GCLM expression in THP-1 cells. ALA and curcumin induce HO-1 expression through p38 MAPK in THP-1 cells [9,10]. EGCG also activates Nrf2 expression in B lymphoblasts via p38 MAP kinase [13]. Here, the p38 MAPK inhibitor SB203580 did not significantly inhibit EGC-induced HO-1 mRNA expression, suggesting this pathway is not involved. EGC activates ERK MAP kinases in Ehrlich ascites tumour cells and HepG2 cells [3,22] and EGCG-induced HO-1 expression in epithelial and endothelial cells is regulated by ERK MAP kinase and PI3K [14,18]. However, we found that inhibitors of these pathways had no effect on EGC-induced HO-1 expression in THP-1 cells.

The present study implicates a role for PKCδ in Nrf2-regulated EGC-induced HO-1 expression. PKC regulates ARE-mediated gene expression in various cell types [10–12]. PKC phosphorylates Nrf2 on Ser40, enabling dissociation from its inhibitor Keap 1 [31]. EGCG activates PKC in neuronal, astroglioma and phaeochromacytoma cells [32–34]. However, PKC inhibitors did not affect EGCG-induced HO-1 expression in endothelial cells [14]. We have previously shown that a classical PKC regulates LPS-induced HO-1 expression in monocytes [11]. However, inhibitors of classical PKC had no significant effect in this study. However, PKCδ inhibitors suppressed EGC-induced HO-1 expression. In addition, Ro-31-8220 and rottlerin, but not Go6976 or LY333531, significantly inhibited EGC-induced GCLM expression, suggesting that PKCδ also regulates EGC-induced GCLM mRNA expression in THP-1 cells. Curcumin-induced HO-1 and GCLM expression are mediated via PKCδδ in human monocytes and THP-1 cells, suggesting a common pathway of activation for these polyphenols. However, p38 also regulates curcumin-induced HO-1 expression in THP-1 cells [10], suggesting some divergence in the two pathways. Based on our studies and other recent reports [10,12,35], PKCδ is emerging as an important member of the signalling pathways leading to Nrf2/ARE-mediated gene expression and its exact role warrants further investigation.

GCLM is the regulatory unit of the rate-limiting enzyme of glutathione synthesis and GCLM induction leads to an increase in glutathione in cells [36]. In the present study, EGC weakly increased GCLM mRNA expression in THP-1 cells. In contrast, EGC decreased glutathione concentrations in Ehrlich ascites tumour cells but this correlated with cell viability [22]. EGC was not cytotoxic at the concentrations used here, suggesting this may be a reason for the differences between the two studies. Interestingly, although NQO1 and ferritin are also regulated by the ARE and green tea extract activates NQO1 in vitro and in vivo [3,4], EGC did not activate these genes in THP-1 cells, suggesting that an alternative catechin may be responsible for the effect of green tea extracts on NQO1 expression or that this effect is cell type-specific.

In conclusion, EGC, in addition to EGCG, contributes to the activation of cellular anti-oxidants by catechins, including HO-1 and this, in turn, may partially be responsible for the beneficial effects of green tea extracts in vitro and in vivo. HO-1 plays a protective role in models of vascular diseases [37,38], suggesting that activation of HO-1 by EGC warrants further investigation. In addition, PKCδ regulates EGC-induced HO-1 expression, confirming that this kinase is emerging as an important member of the signalling pathways leading to HO-1.
